# High-resolution analysis of transmural myocardial perfusion gradients from first pass perfusion MR data. Diagnostic criteria for the detection of coronary artery disease

**DOI:** 10.1186/1532-429X-13-S1-P41

**Published:** 2011-02-02

**Authors:** Amedeo Chiribiri, Gilion Hautvast, Timothy Lockie, Andreas Schuster, Geraint Morton, Shazia Hussain, Marcel Breeuwer, Sven Plein, Eike Nagel

**Affiliations:** 1 King’s College London, Wellcome Trust – EPSRC Centre of Excellence in Medical Engineering, London, UK; 2 Philips Healthcare, Imaging Systems - MR, Best, Netherlands; 3 King’s College London, London, UK; 4 Division of Imaging Sciences - King’s College London, London, UK; 5 King’s College London, NIHR Biomedical Research Centre, London, UK; 6 Philips Healthcare, PCCI Clinical Science & Advanced Development and Eindhoven University, Eindhoven, Netherlands; 7 University of Leeds, Multidisciplinary Cardiovascular Research Centre and Division of Imaging Sciences, King’s College London, Leeds - London, UK

## Objective

To define sensitivity and specificity of thresholded myocardial transmural perfusion gradients in the diagnosis of coronary artery stenosis and to describe the characteristics of perfusion gradients using novel indexes of myocardial perfusion.

## Background

Conventional quantitative assessment of myocardial perfusion analyzes the temporal relation between the arterial input function and the myocardial signal intensity curves, thereby neglecting many of the spatial and temporal relations within the myocardial signal intensity curves. These relations can be described by calculating the perfusion gradient between the left ventricular (LV) epicardial endocardial layers and expressing indexes such as the persistence (seconds), and strength (mean gradient / circumferential extent) of the gradient. As myocardial ischaemia predominantly affects the endocardial myocardial layer due to its higher oxygen demand, transmural gradient indexes may be sensitive markers of ischaemia.

## Methods

11 patients listed for coronary angiography underwent first-pass perfusion imaging at 3T (Philips Achieva, The Netherlands). Perfusion data were acquired in three LV short axis slices with a saturation recovery gradient echo method (TR/TE 3.0ms/1.0ms, flip-angle 15°; effective k-t SENSE acceleration 3.8, spatial resolution 1.2x1.2x10mm) during adenosine-induced hyperaemia (140µg/kg/min) using 0.05mmol/kg Gd-DTPA (Magnevist, Schering, Germany) at 4ml/minute followed by a 20 ml saline flush.

Transmural perfusion gradients were measured for 60 angular positions per slice. Based on results from pilot studies in volunteers, these values were thresholded for values of 5% peak gradient, i.e. a peak endocardial to epicardial gradient at maximal hyperaemia of >5% was considered abnormal. Sensitivity and specificity were calculated comparing the results of segmental gradients and coronary angiography. Correlation between myocardial segments and angiography used the standard 16-segment AHA model.

## Results

At a threshold of 5%, the transmural perfusion gradient analysis yielded a sensitivity of 94% and a specificity of 42% for the detection of significant coronary stenosis on a per-vessel basis. Additional analysis combining the persistence or the strength of the gradient with a 5% threshold resulted in significant differences between positive and negative segments (Table 1) and a sensitivity/specificity of 88%/90%.

**Table 1:** 

	Persistence (seconds)	Strength (% degrees-1)
Positives	17.6±9.6	35.1±23.2
Negatives	3.1±3.9	5.3±5.1
T-test	0.002	0.008

## Conclusions

Transmural perfusion gradient analysis allows for a sensitive identification of ischaemic coronary artery territories. The additional analysis of derived transmural perfusion indexes improves the specificity of the analysis by allowing the identification of small and non-significant perfusion gradients.

